# Novel *GPR156* variants confirm its role in moderate sensorineural hearing loss

**DOI:** 10.1038/s41598-023-44259-4

**Published:** 2023-10-09

**Authors:** Memoona Ramzan, Nazim Bozan, Serhat Seyhan, Mohammad Faraz Zafeer, Aburrahman Ayral, Duygu Duman, Guney Bademci, Mustafa Tekin

**Affiliations:** 1 John P. Hussman Institute for Human Genomics, University of Miami Miller School of Medicine, 1501 NW 10Th Avenue, BRB-610 (M860), Miami, FL 33136 USA; 2 Department of Otolaryngology, Yuzuncu Yil University School of Medicine, Van, Turkey; 3 Department of Medical Genetics, Faculty of Medicine, Uskudar University, Istanbul, Turkey; 4 Department of Audiology, Ankara University Faculty of Health Sciences, Ankara, Turkey; 5 Dr. John T. Macdonald Foundation Department of Human Genetics, University of Miami Miller School of Medicine, Miami, FL USA

**Keywords:** Genetics, DNA, Genetics research

## Abstract

Hereditary hearing loss (HL) is a genetically heterogeneous disorder affecting people worldwide. The implementation of advanced sequencing technologies has significantly contributed to the identification of novel genes involved in HL. In this study, probands of two Turkish families with non-syndromic moderate HL were subjected to exome sequencing. The data analysis identified the c.600G > A (p.Thr200Thr) and c.1863dupG (p.His622fs) variants in *GPR156*, which co-segregated with the phenotype as an autosomal recessive trait in the respective families. The in silico predictions and a minigene assay showed that the c.600G > A variant disrupts mRNA splicing. This gene belongs to the family of G protein-coupled receptors whose function is not well established in the inner ear. *GPR156* variants have very recently been reported to cause HL in three families. Our study from a different ethnic background confirms *GPR156* as a bona fide gene involved in HL in humans. Further investigation towards the understanding of the role of GPCRs in the inner ear is warranted.

## Introduction

Normal hearing is broadly dependent on well-organized intricate structures called hair cells. Hair cells are the core part of the inner ear, which is responsible for converting sound signals to electrical signals that are later transmitted to the brain^1^. The apical portion of hair cells have thin finger-like projections called stereocilia which play the role of mechanosensation and fluid motion around the hair cells^2^. Disruption of stereocilia is a quite common phenomenon observed in various hearing loss (HL) disorders^3^. In a recent report, mutations in *GPR156,* encoding one of the G proteins coupled receptor (GPCR) localized to hair cell stereocilia were shown to cause HL in three unrelated families^4^.

GPCRs are classified into 5 groups: Rhodopsin, Secretin, Adhesion, Glutamate, and Frizzled/ Taste2^5^. All GPCRs have seven conserved helices spanning the membranes of respective cells. These helices undergo conformational changes after binding with certain ligands (Gα, Gβ, Gγ) and hence start the signaling processes^6^. Several GPCRs have been identified in the cochlea pointing out their essential role in this organ. However, only a few are well-studied in human and model organisms.

We are presenting findings from two unrelated Turkish families which were enrolled in a large study comprised of a multiethnic cohort of 322 families. The goal of this study was to identify causative gene variants in these families in which multiple members were presented with non-syndromic HL. Two novel variants in *GPR156* were identified in respective families which has confirmed its role to cause non-syndromic moderate HL in a different population.

## Methods

### Ascertainment and audiological assessment

This study was approved by Institutional Review Board at University of Miami, USA and Ankara University Medical School Ethics Committee, Turkey. All the experiments were performed in accordance with the relevant guidelines and regulations. Probands of family 2254 and 2986 were identified at audiological and ENT clinics in Turkey. Written informed consents were obtained from participants or their legal guardians to participate in a large study on genetics of HL being conducted at University of Miami. The audiological exam was performed to measure average hearing thresholds for all the participants at frequencies of 0.5, 1, 2, 4 and 8 kHz in soundproof rooms and Pure Tone Averages (PTAs) were calculated. CT scan or MRI were performed to check any anomalies of the temporal bone. Romberg and Tandem Gait tests were completed to identify vestibular defects.

### Exome sequencing (ES) and variants filtering

DNA samples taken from both families were first screened via Sanger sequencing for the presence of any variation in *GJB2*, the most common gene for HL. The probands from each family were subjected to Exome sequencing (ES). Exome capture was performed using IDT xGen V2 at BGI and sequencing was completed at 100X paired end on HiSeq 2000 platform. Reads were aligned with BWA mem 0.7.8 to the1000 genomes phase 2 (GRCh37) human genome reference sequence and variant calling was performed using GATK (https://www.broadinstitute.org/gatk/). The final output was obtained in the form of FASTQ files and uploaded to an in-house software GENESIS (https://app.tgp-foundation.org) for variant analysis. As a first step the data was filtered against the population frequencies in the genome Aggregation Database (gnomAD) and variants with allele frequency of less than 0.0007 for autosomal recessive disorders as prescribed by HL-EP were retained^7^. All the homozygous, hemizygous and compound heterozygous exonic and splice site variants present in known deafness genes were carefully examined. The damaging effects of variants were analyzed using in-silico pathogenicity scores from various tools such as SIFT, PolyPhen-2, MutationTaster, REVEL (http://sites.google.com/site/revelgenomics/), CADD and MAVERICK (https://www.researchsquare.com/article/rs-1602211/v1). MAVERICK scores are helpful to predict pattern of inheritance along with the pathogenicity of a variant. It uses AI to classify variants based on their impact on the gene product and reported variations of a given gene. It was shown that a benign score > 0.5 can be helpful to remove as much as 98% of benign variants from the data which led to the most refined results. Additionally, the pathogenicity for splice variants was also predicted using ASSP (http://wangcomputing.com/assp/), Varseak (https://varseak.bio/), MaxEntScan and BDGP (https://fruitfly.org/seq_tools/splice.html). The splice predictions were also examined using dbscSNV Ada/RF scores given in Franklin (https://franklin.genoox.com/clinical-db/). dbscSNV Ada/RF predicts pathogenicity of Single Nucleotide Variants (SNVs) within splicing consensus regions ranging from − 3 to + 8 at the 5′ splice site and − 12 to + 2 at the 3′ splice site. These tools predict the potential of a splice altering variant by using ensemble score computed using AdaBoost or Random Forest algorithm on the outputs of other prediction tools. The scores range from 0 to 1, where higher values demonstrate the deleterious effect. Copy Number Variations (CNV) were detected using CoNIFER v.02.2 with default parameters.

The potential rare pathogenic variants identified in each proband were tested for segregation in the complete family using Sanger sequencing.

### Functional characterization of the c.600G > A variant in* GPR156*

To validate the disruptive splicing effect of synonymous variant c.600G > A (exon 6), a minigene assay was performed. Briefly, a 966 bp region of wild type and mutant genomic DNA containing exon 6 and flanking introns were amplified using primers with overhangs for Xho1 and SacII restriction enzymes. The amplified PCR products were cloned into pET01 minigene vector (MoBiTech, Germany) endowed with an intrinsic splicing system. The resulting constructs were transformed, and colonies were selected on ampicillin (100 µg/mL) plates. The recombinant plasmids were confirmed by Sanger sequencing using the primers provided by the manufacturer. The confirmed wild type and mutant minigenes as well as pET01 plasmid were transfected into HEK293 cells and RNA was isolated after 48 h. cDNA was synthesized using SuperScript™ IV First-Strand Synthesis System (Thermo Fisher, USA) and cDNA primer specific to pET01 provided along the kit. The cDNA was PCR amplified with primers SD-F (GATGGATCCGCTTCCTGCCCC) and SA-R (CCGTGACCTCCACCGGGCCCTC) supplied by the manufacturer and the resulting products were isolated after electrophoresis using 2% agarose gel. The gel bands were excised, and the purified DNA was verified by Sanger sequencing.

## Results

### Clinical evaluation

**Figure 1:**
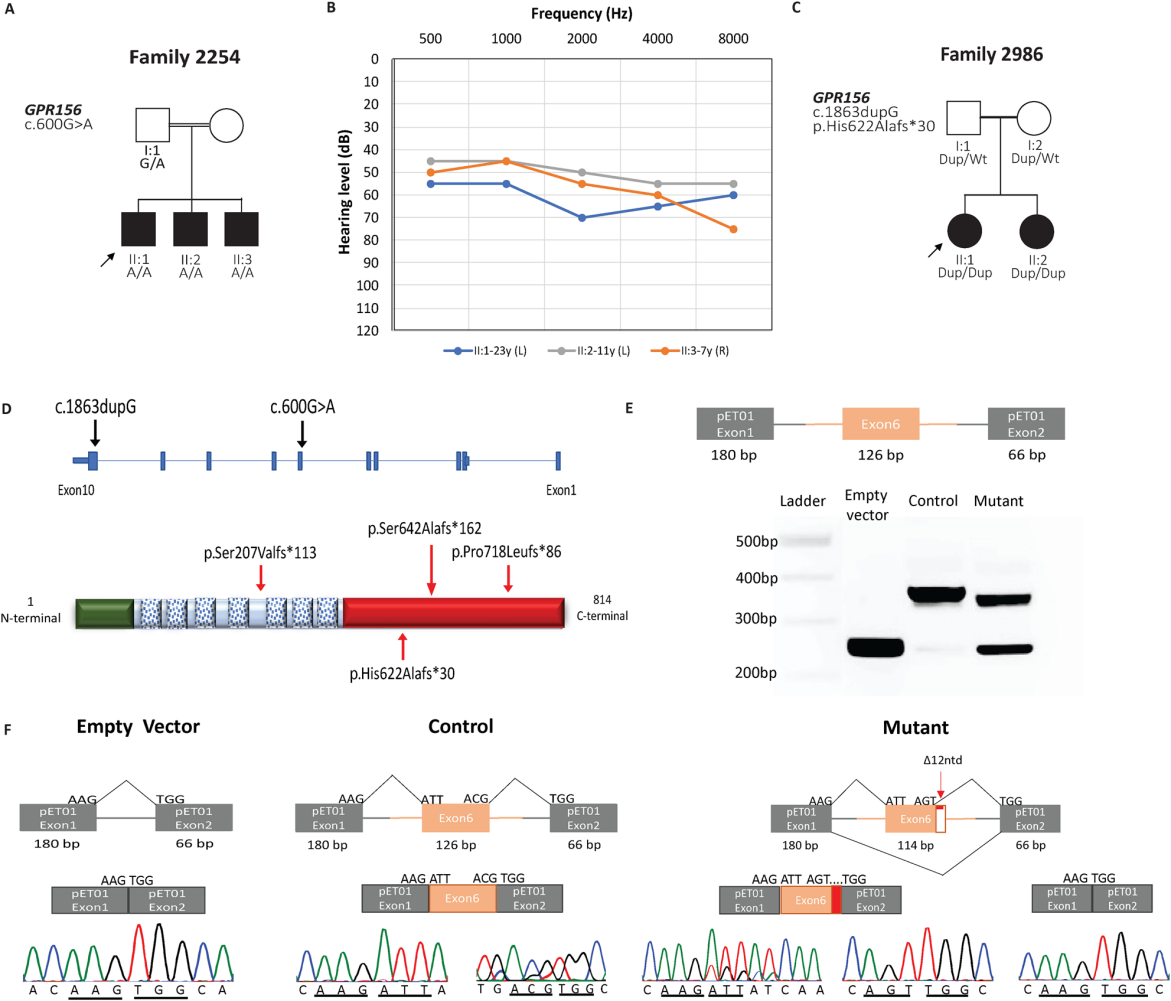
Pedigrees of families 2254 and 2986, pure tone audiometry and detail of identified and reported variants. (**A**) Pedigree of family 2254 showing co-segregation of c.600G > A in *GPR156* with hearing loss. Shaded symbols represent affected individuals and double lines indicate consanguineous marriage. Arrow indicates proband for which exome sequencing was performed. (**B**) Audiograms from better hearing ears of individuals II:1, II:2 and II:3 showing moderate hearing loss. (**C**) Pedigree of family 2986. The variant c.1863dupG co-segregates in the family with hearing loss. (**D**) Schematic representation of *GPR156* (NM_153002.3) gene and encoded protein (NP_694547.2). *GPR156* consists of a total of 10 exons and the novel variants identified are present in exon 6/10 and 10/10. The encoded functional protein is comprised of 814 amino acids. It has a small extracellular region (green, amino acids 1–47), 7 coiled coil regions (21 amino acids each) present in the membrane and a long cytoplasmic tail (red, amino acids 310–814). The novel variants are shown at the bottom and reported ones at top. (**E**,**F**) *GPR156* exon 6 inserted into the pET01 exontrap vector. Gel electrophoresis after PCR amplification of extracted cDNA from HEK293 cells transfected with empty vector, a wild type and mutant construct. The Sanger sequencing showed the deletion of 12 nucleotides (red arrow) and skipping of exon 6 in the mutant construct. The traces for exon-to-exon boundaries are underlined.

Family 2254 has 3 affected males (Fig. 1A) with ages 23, 11, and 7 years, born to consanguineous parents. All siblings presented with congenital or prelingual onset sensorineural HL. The PTAs showed that all the three affected individuals have moderate HL (II:1 49 dB, II:2 53 dB and II:3 61 dB) (Fig. 1B). Besides that, physical examination and laboratory evaluations suggested non-syndromic HL in this family. Temporal MRI scans were normal and Romberg and Tandem gait tests indicated normal vestibular function. The family was recontacted and re-examined after 5 years for any changes in hearing thresholds. The HL was stable, and individuals did not have additional abnormalities except for the youngest individual (II:3) who became myopic.

Family 2986 includes two affected sisters aged 17 and 9 years (Fig. 1C). Both sisters have non-syndromic bilateral moderate sensorineural HL (II:1 41 dB and II:2 51 dB). The elder sister (II:1) was diagnosed with HL at the age of 8. The younger sibling (II:2) has bilateral prelingual onset HL which was diagnosed and managed at an earlier age. Physical examination and medical history revealed no other clinical phenotype related to HL in this family. Temporal bone CT scans were normal. There was no vestibular abnormality. Both affected sisters were diagnosed with myopia in addition to HL.

### Genetic findings

ES data for proband II:1 from family 2254 was analyzed. He was found to be homozygous for a synonymous variant c.600G > A (p.Thr200Thr) in *GPR156* (NM_153002.3) (Table 1), which co-segregated with HL in the family after Sanger sequencing (Fig. S1). All the affected individuals were homozygous for the variant and unaffected father was a healthy carrier (Fig. 1A and Fig. S1).

**Table1:** Identified causative variants in *GPR156* which co-segregated with phenotype in respective families.

Family ID		Family 2254	Family 2986
Variant		NM_153002.3	NM_153002.3
		c.600G > A	c.1863dupG
		p.Thr200Thr	p.His622Alafs*30
gnomAD frequency		0.00003	Absent
Internal control frequency (Turkish)		0/1612	0/1612
MaxEntScan	Score	9.2	N/A
MAVERICK (AR)	Score	N/A	0.5
TraP	Score	0.97	N/A

*MaxEntScan* maximum entropy scan, *MAVERICK* mendelian approach to variant effect pRedICtion built in Keras, *TraP* transcript-inferred pathogenicity score.

The variant c.600G > A appeared as a synonymous change, which alters the last nucleotide of exon 6 (Fig. 1D). The splicing prediction tools ASSP and BDGP predicted the loss of authentic donor splice . Additionally, high scores from Varseak (ranked as class 5 variant affecting splice site), MaxEntScan (9.2), dbscSNV Ada/RF (1 and 0.98 by Ada and RF algorithms) and TraP (0.97) suggested c.600G > A as a splice disrupting variant. The minigene assay showed that this variant leads to the skipping of entire exon 6 in the majority of transcripts and also to an in-frame deletion of 12 nucleotides in exon 6 in a minority of transcripts (Fig. 1E,F and Fig. S2).

In addition to *GPR156* there were 3 other rare loss-of-function and missense variants in genes which were not previously associated with HL (Table S1 and Fig. S1). All three of these failed segregation tests in the family.

In family 2986, a duplication c.1863dupG in *GPR156* leading to frameshift was identified in the proband II:1 (Table 1), which co-segregated in the family with the phenotype (Fig. 1A). We identified that beside *GPR156*, the proband was homozygous for nonsense variants in *ALPPL2*, *ABTB2* and *DDX31* (Table S1). The segregation analysis confirmed c.1863dupG in *GPR156* as only causative gene in this family (Fig. S1). The identified variants in *GPR156* in both families were absent from internal control database containing 1612 Turkish samples and are extremely rare (MAF = 0.00003) or absent in other population databases.

## Discussion

G protein coupled receptors (GPCR) are known to have an important role in several physiological processes crucial for normal functioning of a human body^8–10^. In recent decades researchers have shown that more than 30 GPCR are expressed in cochlea in different mouse models^10^. The accurate function of this huge number of GPCRs expressed in the cochlea is yet to be determined. So far, the role of three GPCRs; ADGRV1, SIP2 and GPR126 has been studied in humans, mouse, and zebrafish^11–13^. ADGRV1 is required for hair cell polarity, maturation, and organization^14,15^ while *gpr126* is known for the proper formation of semicircular canals in zebrafish^13^. Whereas, SIP2 and the respective receptor has been studied for its role in cisplatin induced HL^16^.

International Mouse Phenotyping Consortium (IMPC) has published a few phenotypes possibly associated with *Gpr156* in a mouse model (*Gpr156*^*del/del*^). Auditory phenotype was not found to be significantly different in those mice, even though Auditory Brainstem Response (ABR) traces appear to be abnormal. In a different study, the same mouse model was found to have HL by ABR and distortion product otoacoustic emissions (DPOAEs) in P30 animals. Histology evaluation showed a graded range of misorientation (inverted 180° relative to controls) across rows of outer and inner hair cells. HC misorientation and morphological defects were observed at both early (E17.5) and mature stages (P30) in those mice^17^.

The results present in the above study were emphasized by the discovery of *GPR156* variants in three human pedigrees associated with congenital deafness^4^. The audiograms were provided for only two affected individuals (ages 6 and 8 years) which showed moderate HL. However, this report does not provide any conclusive evidence regarding stability and severity of HL phenotype in all affected participants.

*GPR156* is highly expressed in the apical region of HC and the reported loss of function variants (p.Ser207Valfs*113, p.Ser642Alafs*162 and p.Pro718Leufs*86) (Fig. 1D) cause either nonsense mediated decay or the production of non-functional truncated protein^4^. Similarly, we report on two additional families with prelingual-onset moderate HL co-segregating a frameshift (exon 10/10) and splice (exon 6/10) variants. Likewise, the reported variants may also produce non-functional proteins leading to HL in these families.

Since many GPCRs are expressed in the inner ear and only a few of them have been associated with HL, there is need to explore the role of these receptors in the mechanism of audition. This ultimately can be helpful in solving the mystery of hearing mechanism and management of HL. Identification of additional families with pathogenic variants in *GPR156* has emphasized the role of this gene in hearing.

### Supplementary Information

Supplementary Information.

## Data Availability

All data generated or analyzed during this study are included in this article and its supplementary information files. The genetic variants are submitted to ClinVar database.
