# Supplementary material for: Novel GPR156 variants confirm its role in moderate sensorineural hearing loss
Source: Sci Rep. 2023 Oct 9;13:17010. doi: 10.1038/s41598-023-44259-4 (PMC10562426; doi:10.1038/s41598-023-44259-4)
Supplement: Supplementary file 1 — Supplementary Information. [file 41598_2023_44259_MOESM1_ESM.pdf]

Table S1: List of other potentially causative variants tested in probands of families 2254 and 2986 after Exome sequencing.

Chromosomal position	Gene	HGVS variant	Variant type	Pathogenicity scores				Inner ear expression
				CADD	REVEL	MAVERICK (AR)	GERP	
**Homozygous variants identified in individual II:1, family 2254**								
Chr20:20033266	*CRNKL1*	NM_016652.6 c.204C>A p.Cys68*	Ns	n/a	n/a	0.76	n/a	IHC, OHC, PC, DC
Chr3:121345549	*FBXO40*	NM_016298.4 c.1922A>G p.Gln641Arg	Ms	22	0.1	0.68	6.1	IHC, OHC, DC
Chr17:37902208	*GRB7*	NM_005310.5 c.1382G>A p.Arg461His	Ms	29.8	0.86	0.97	5.2	IHC, OHC, PC, DC
**Homozygous variants identified in individual II:1, family 2986**								
Chr11:34379063	*ABTB2*	NM_145804.3 c.67delG p.Ala23Profs*39	Fs	n/a	n/a	0.68	n/a	IHC, OHC, PC, DC
Chr2:233274079	ALPPL2	NM_031313.3 c.1221C>G p. Tyr407*	Ns	n/a	n/a	0.99	n/a	none
Chr9:135536600	DDX31	NM_022779.8 c.762G>T p. Met254Ile	Ms	24.8	0.1	0.8	5.7	IHC, OHC, PC, DC

Chromosomal positions are mentioned according to GRch37/hg19 reference assembly. Inner ear expression was analyzed using gEAR portal gene expression database.

MAVERICK score for autosomal recessive inheritance pattern of the respective gene is given to assess the pathogenicity.

Ms: Missense, Ns: Nonsense, Fs: Frameshift

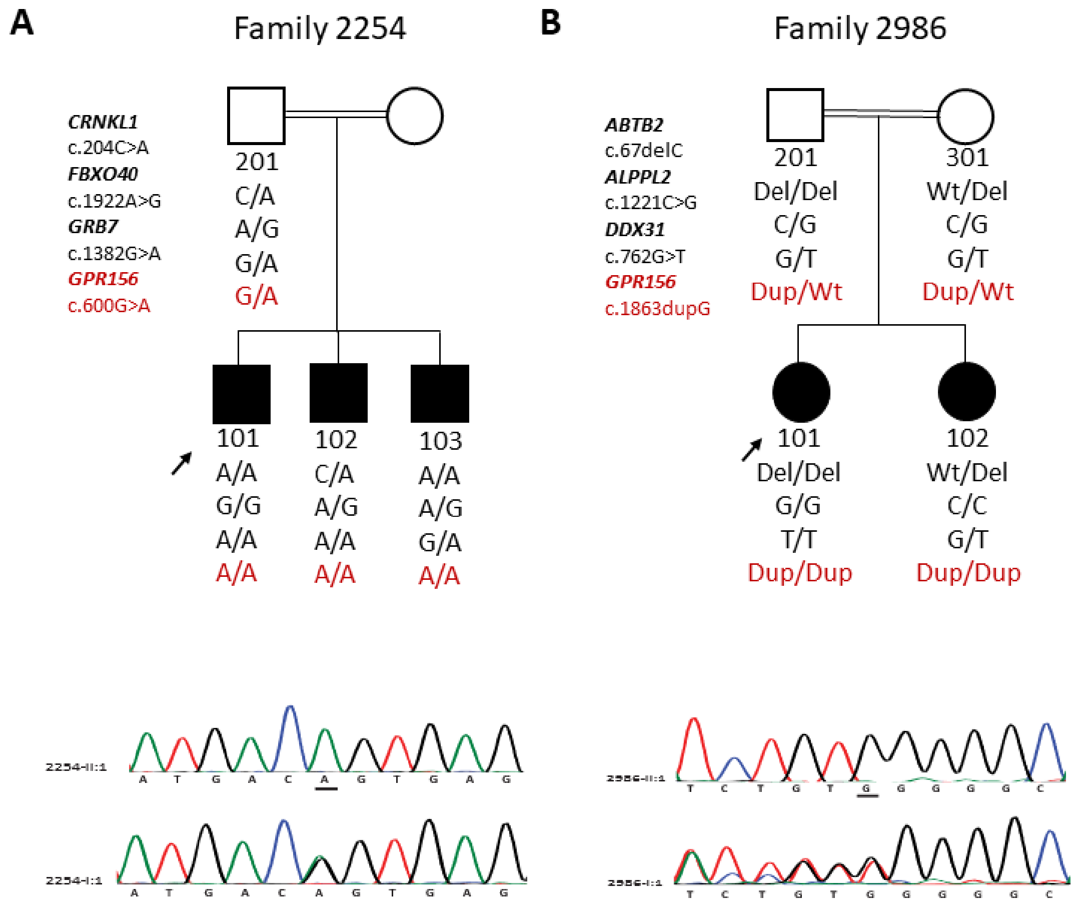

Figure S1: A & B) Segregation analysis of additional rare homozygous variants along with those in *GPR156* identified in families 2254 and 2986. Exome sequencing was performed for the proband (marked with arrow) from each family. The partial sequence traces below each pedigree represents co-segregation of *GPR156* variants (c.600G>A and c.1863dupG) in both families. The mutated residue is underlined.

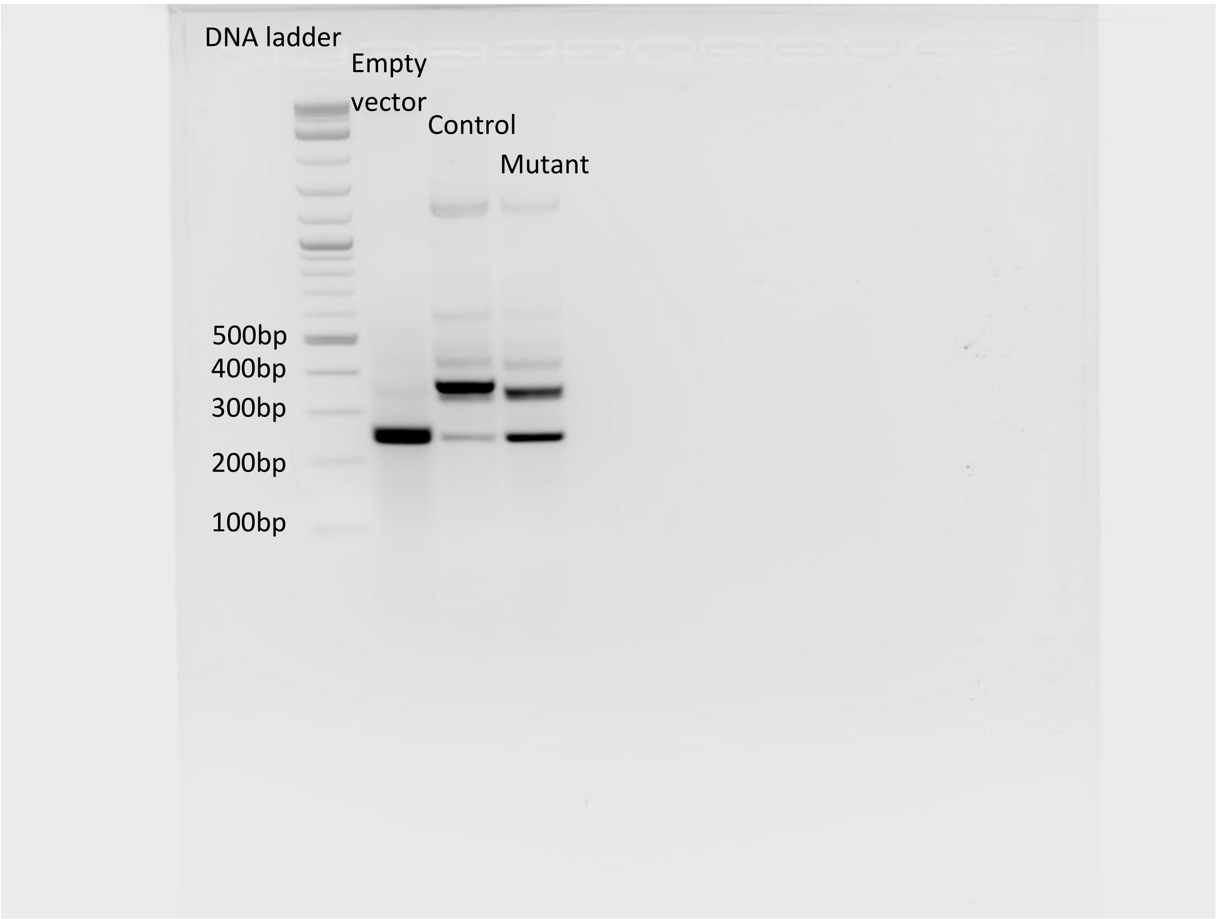

Figure S2: Uncropped gel image representing bands after PCR amplification of cDNA from HEK293T cells transfected with an empty pET01 vector, wild type and mutant minigenes.
